# Selections from Foreign Periodicals

**Published:** 1887-01

**Authors:** 


### SELECTIONS FROM FOREIGN PERIODICALS.

POISONING OF HORSES BY THE INDIAN PEA, "*Lathyrus Sativus.*" Condensed from the lectures of Professor James McCall, Principal Veterinary College, Glasgow.

In considering the diseases which attack the larynx and lead to roaring in horses, attention was called to a specific form of the disease produced by the vetch or pea imported from India.

The animals attacked presented the following symptoms: difficult breathing, each respiratory movement being accompanied by a loud sound which seemed to come entirely from the larynx; pulse quick, irregular and intermittent; profuse perspiration; temperature normal; death generally occurring suddenly by "Apnœa."

*Post-mortem* examination revealed conjestion of the mucous membranes, especially of larynx and stomach, with serous exudation beneath and an accumulation of mucus and blood mixed with air in the bronchial tubes. The ganglionic of the motor cornua of the spinal cord were diminished in number and atrophied.

The cause of the disease was found to exist in the food, as only those horses were attacked which had been fed on a mixture containing Indian peas.

The toxic agent seems to have a direct influence on the nervous system, having the power of disturbing the function of the nerve-centers, especially those which control the circulation and respiration, its continued action resulting ultimately in organic disease, not only of the nerve-centers themselves, but also of the organs supplied by them.

No bacillus is found in the blood of the affected animals, although Prof. Williams is inclined to believe that the dirt surrounding the pea contains some variety of micro-organism. This latter theory is contradicted by the fact that no microbes are to be found on microscopic examination and that there is no increase of temperature. Moreover, change of diet arrests the disease which would not happen if these bacteria once entered the circulation.

The *Lathyrus Sativus*, then contains a poison, and this poison acts especially on the recurrent branches of the pneumo-gastric nerve.

J. O'Leary, M.D.

Thrombosis of the Iliac Arteries, complicated with Arteritis, Embolism and Gangrene. By M. M. Cadeac and Maley.

In veterinary medicine, the case of gangrene following the stoppage of circulation in a limb in consequence of arterial plugging, has not been hitherto observed. Although the occurrence has long been considered possible, yet a case has not been described. On an old horse, put aside for surgical operations, the above authors have found the lower part of the off hind limb œdematous and cold ; the hair about these parts could be removed in handfuls.

Autopsy demonstrated that this moist gangrene of the whole of one extremity was due to the destruction of the iliac arteries, external and internal of the right side, by an old blood-clot, stratified and adherent, which was prolonged into all the divisions of the above arteries, without completely obstructing them, and being broken at different points.

Microscopic examination showed that the finest arteries were plugged by the blood-clot. The internal coat of the vessels plainly showed the changes wrought by inflammation, of old standing in some points and more recent in others. In consequence of stagnation of blood in the vessels hemorrhages were produced, principally in the muscular interstices of the upper part of the limb. The authors have not been able to note the complete symptoms of this case, but, such as it is, their observation merits record.—*Revue Veterinaire*, November, 1885.

(*b*). *Aneurism of the Aorta and Endocarditis Chronica.*—Wolff found in a stud-bred horse, twenty years old, the posterior aorta from its origin, 40 c.m. in length, all in knots. The knots were of three sizes. The tricuspid valves were much thickened, and the heart was hypertrophied. No interference of the circulation, as a result of these knots, could be detected during life.

(*c*). Guckel describes an enlargement in the neighborhood of the kidneys, in an ox, which was fully the size of a man's head, and was surrounded by a yellowish-colored membrane. The swelling weighed twelve pounds. On section the swelling showed several large blood-vessels, in which two fingers could be easily introduced at a time, and which ended in blind sacs. The smaller sacculations contained a dark-red, gritty blood. The membrane surrounding the enlargement was also of a dark-red, hæmorrhagic appearance. Many of the vascular sacculations, in the interior of the growth, were in connection with the vena cava posterior.—*Archiv fur Wissen. u. Praktische Theirheilkunde, Band XII., 3 und 4 Heft, 1886.*

(*d*). *Beri-Beri.*—Professor Lacerda (Report to the Academy of Medicine, 29th January, 1884) practised inoculation of a cultivation of blood from the tongue of Beri-beri patients in beef-tea (*sterilised*). This beef-tea, on being examined, showed the evolution of certain microbes. Inoculation of this broth of cultured microbes killed rabbits more or less rapidly, and their organs were full of the same microbes, i.e., *bacilli.* He noticed that large quantities of bad rice had been eaten by a Beri-beri patient, and immediately examined the rice and found it the dwelling-place of the same microbes which he had found in the bood of patients suffering from Beri-beri. Rats that ate the rice died, and their organs contained the bacilli.

Kœniger (Archives for Clinical Medicine, 1884) attributes the disease to miserable food and sanitary arrangements. Mendes (Contributions to the Study of Beri-beri, 1884) also found microbes in the dilated blood-vessels of the brain and cord of patients suffering from Beri-beri. Lodewijks and Wiess mention atheroma of the blood-vessels and of the heart as a frequent complication of the disease. Baels (Seitschrift f. Clin. Med., 1882) describes multiple peripheral neuritis as a frequent complication of Beri-beri.

Ballet (Societe Anatomique, July, 1883) describes a form of paralysis consecutive to Beri-beri.

Morehead, a great Indian authority, asserted the similarity between Beri-beri and scurvy.

Dr. Wallace Taylor has satisfied himself that Beri-beri is due to the presence of a bacterium.

Dr. M. Ogata, of the Medical Department of the Japanese Imperial University, has carried out experiments on the bacterial origin of Beri-beri, or *kakke*, for the last four years. Dr. Ogata comes to the conclusion (*Official Gazette*, April 13th, 1886) that the bacterium present in *kakke* may be cultivated and distinguished by its behaviour under cultivation; that it causes symptoms of *kakke* in monkeys, dogs, rabbits and other animals ; that an acid substance, obtained during the development of the bacteria in nutrient fluids, causes paralysis of the hind limbs in animals. The poison enters chiefly through the digestive system. Dr. Wallace Taylor found that, in 134 cases, the average richness of blood corpuscles was 94 per cent. of the normal. Of the coloring matter (hæmoglobin) of the blood, he found 81 per cent., the amount being sometimes as low as 65 per cent. He, however, finds no relation between the severity of the disease and the proportion of hæmoglobin. Marked anæmia in Beri-beri is met with only in cases complicated with digestive or other derangements. Dr. Wallace Taylor frequently found that subjects of Beri-beri had been made so much worse by being sent to the hills for a change, that he now never gives his consent to the moving of patients where cardiac debility is marked, as weakness of the heart is the chief cause of mortality in this disease. Dr. Wallace Taylor was the first to suggest that Beri-beri was similar, in many respects, to the common diseases, known in India and some parts of Burmah under the name of *Kumri.* There is a chronic form of Beri-beri, which, he suggests, is still more closely related to Kumri than the acute form. The bacterium of Beri-beri is not found in the blood of patients suffering from Kumri, and whilst Beri-beri is generally acute and only occasionally chronic, Kumri, on the other hand, is generally chronic and rarely acute.

R. W. BURKE, V. S.

VETERINARY QUACKERY.—An interesting result of veterinary quackery is reported in the *Oesterreichische Vierteljahrschrift fur Veterinar Kunde* (1885, X, p. 187). Dr. Swaty met a countryman on the road whose horse had a leather bandage on the right side of the head. He induced the man to remove it, and to his surprise found both the greater and lesser sinuses (antrum of Highmore) of the right maxilla *open!* The nasal cavity was also partly exposed. On inquiring, he learned that the horse had been purchased in 1859 as a yearling. In January, 1861, warts developed at various points, several being situated on the face. To remove these the owner used an ointment given him by a quack. The warts were incised and the ointment "generously" applied. The result was an intense inflammation, and two weeks later the warts, together with the underlying skin and the bony lamella, fell off. The margins of the gap thus produced healed rapidly. With this enormous defect, which was protected by the leather bandage referred to, and carefully cleansed every fortnight, the poor horse *worked twenty-four years in good health* without any disturbance of health, and, most remarkable of all, no local disturbance of the exposed mucous membranes. The destroyed bone included part of the lachrymal, upper maxilla and nasal bones, as well as the upper meatus. The right frontal sinus could be seen into from the gap.

E. C. SPITZKA, M. D.

THE ETIOLOGY OF GLANDERS,* by Dr. Loefler.

The existence of glanders or glanders-farcy, was known to the ancients, and Hippocrates has left us a description of its symptoms characterized by the truthfulness and realism which stamp all the writings of the Father of Medicine. The question of the contagiousness of the disease was long under discussion and was not definitely settled until the beginning of the present century, when experiments conducted simultaneously in Germany, England, and France placed the matter beyond dispute so that now it is universally conceded that the characteristic secretion of the disease is eminently contagious. The investigations of Chauveau in France, and Hallier in Germany, have established not only the parasitic

* Arbeiten aus dem Kaiserlichten Gesundheitsamte. Vol. I. Berlin. 1886.

origin of glanders, but point to the identity of its virus with that of syphilis. Concerning this supposed identity of the two poisons, however, later and more reliable researches have contributed no positive assurance. The experiments of Koch have proved in addition to the fact that glanders are the product of micro-organisms residing in a congenial medium, the equally interesting fact that the spores of farcy are distinctly *sui generis*, and thus constitute it a contagious disease of a different nature from tuberculosis. The researches of Claude Bernard had already inspired the belief that this was the case, but the more careful and scientific methods of Koch threw such light on the matter that these points are now regarded as placed beyond the pale of dispute. Touching the liability of other animals than the horse to contract the disease from exposure to contagious influences, it has been ascertained that the goat and the cat evince an extraordinary degree of susceptibility to the poison of glanders and exhibit *post mortem* evidences of the virulent activity of the disease. The ruminants enjoy almost complete immunity from the contagious action of glanders, though inoculation has been attended in many cases with eruptions and some constitutional disturbance, but in no instance sufficient to warrant the view that the disease had been communicated. Experiments made upon the dog prove at least that the animal is not a fit subject for inoculation with the virus of glanders, since the train of symptoms developed thereby, though grave and in some instances fatal, have given no evidence that these were caused by a specific poison. The practical conclusion to be deduced from the most recent experiments made in connection with the disease are that prophylaxis of the most vigilant kind can alone arrest the spread of this destructive disorder, and that it is better to sacrifice the lives even of suspected animals than to incur the risk of propagating the disease by the employment of therapeutical measures of questionable value.

C. M. O'Leary, M. D.

Hæmoglobinuria from Cold in the Horse and its Relation to that of Man, by Dr. Floriano Brazzola, in *La Clinica Veterinaria*, July and August, 1886.

Notwithstanding the time and labor devoted to the study of Hæmoglobinuria, its pathogenesis is very obscure, and this is especially so in regard to the form under consideration.

Difference of opinion as to its nature exists, the lesion being thought to be renal by some, spinal by others; many think it consists in an altered condition of the blood, dyscrasis, while in the opinion of a few it is nothing more than a rheumatic affection of the muscular system.

The symptoms as described by Friedberger and Frohner are as follows: the animal is attacked on being taken from a warm and badly ventilated stable and placed in service without having any change made in his food, the attack resembling acute rheumatism with partial paralysis posteriorly and trembling gait and impaired sensibility. The urine is scant and high colored. The spectroscope reveals coloring matter of the blood, but no blood globules, or very few, are to be found under the microscope. Chemical examination shows a large quantity of albumen. Pulse and respiration frequent. Peristaltic movement of intestines diminished or nearly suspended. One attack predisposes to another.

The anatomical lesions according to Bollinger are: thick, black blood, pale muscles, clay colored liver, flacid kidneys, increased spinal subarachnoid liquid, and injected vessels in spinal meninges, especially in lumbar region.

After numerous observations, I have noted the following facts:

The disease declares itself suddenly on exposure of the animal to a low temperature, which acts on the cutaneous surface, the symptoms being restricted movements, profuse perspiration, tremblings, vague colicy pains, paralysis of posterior extremities, hæmoglobinuria and albuminuria. The fever is limited at first, reaching a high grade only during the last days. The pulse is small and frequent, heart beat confused, dyspnœa urgent, and slight gastro-enteric catarrh. The cause of death is profound degeneration, especially of the cardiac muscle, and slow poisoning by carbonic acid.

Autopsy revealed dark diffluent blood with leucocythacmia and destruction of red globules, degeneration of the muscles, hyperæmia of the tissues, œdema and softening of the spinal cord.

Many authors claim that the essence of the disease consists in a primary inflammation of the kidneys. Among them may be mentioned Hofer, Achaty, Meyer, Brückmuller and Zundel; also Clement, in the *Journal of Comparative Medicine and Surgery.* The writer, without denying the possibility of a true nephritis, which, however, is always secondary, thinks that the lesions described by them cannot be ascribed to a true inflammatory process, and cites cases in which the symptoms developed themselves rapidly and death took place without any renal lesion being discovered.

The endeavor to identify this disease with Bright's disease in man is absurd.

Opinions differed as to the pathogenesis of Bright's disease since Bright himself, in 1827, recognized the existence of dropsy dependent on renal affection, accompanied by albuminuria.

Frerichs, in 1851, announced the lesion to be a peculiar process which commenced with hyperamia, followed by exudation and degeneration of the parenchyna and terminating in renal atrophy.

Kleps, in 1870, identified it with a primary interstitial nephritis.

Virchow, in 1871, distinguished three forms; parenchymal nephritis, interstitial nephritis and amyloid degeneration.

Thus we see that no relation exists between Bright's disease and the one under consideration, neither from the physio-pathological nor from the anatomo-pathological point of view.

A second series of authors admit the essence of the disease to be an alteration of the blood secondary to an introduction of an infective substance, thus: Zundel, Lausch, Lowoch and Hering admitted an alteration of the blood and kidneys.

Spinola, Vogel, Haubner, but especially Bollinger, were positive in ascribing it to a primitive alteration of the blood. The latter thinks the essence of the disease consists in this condition leading to hæmoglobinuria, acute nephritis, with albuminuria, œdema, paralysis of the lower extremities. To sustain his view he refers to the observations of various authors who noted the alteration of the blood, muscles and liver, which proved the existence of a septic material in the blood. He does not recognize cold as a cause of the disease.

Vogel, also, in his latest communications, comes to the same conclusion and thinks that the germ of the disease is generated in the stable.

The writer admits an altered condition of blood as the explanation of hæmoglobinuria only, not of the other symptoms, and does not agree with Bollinger, who considers the presence of hæmoglobinuria in the urine as the pathognomonic symptom. According to Ponfick, hæmoglobinuria should be the term applied to the disease.

Frank recognizes the disease as a spinal paralysis, with secondary paralysis of the renal nerves; Friedberger admits a hyperæmia of the spinal cord and membranes; Ableitner and Koniger think it a rheumatic inflammation of the lumbo-sacral portion of the cord; Göring, as a congestion and Csokor as anæmia of the same.

In my opinion, the alteration of the central nervous system cannot be recognized as essential, as it might be only a manifestation of hyperæmia anæmia or inflammation of the medulla spinalis.

In conclusion, I agree with Murri that the pathological process consists in a condition of abnormal excitability, caused by the action of cold on the cutaneous nerves reflecting on the vaso-motor fibers, followed by increase of caliber of the blood vessels, slackening of the circulation and diffuse stasis. This explains the clinical and anatomo-pathological picture already given. *Hæmoglobinuria periodica* of man and this morbid condition in the horse are closely related, both as regards their essence and their pathogenesis.

J. O'Leary, M.D.

